# 

**Published:** 1861-09

**Authors:** 


### CONTENTS.---SEPTEMBER, 1861.

### ORIGINAL CONTRIBUTIONS.

PAGE

ART. I. Lectures on Military Surgery. By E. ANDREWS, A. M., M. D., Professor of Surgery, etc. Lecture V.......... 449

ART. II. Case of Extra-Uterine Pregnancy, Continuing Four Years; Fœtus Removed by Gastrotomy; Recovery. By E. P. COOK, M. D., Mendota, Ill.,.......... 458

ART. III. Action of Opium on the Genito-Urinary Organs. By B. WOODWARD, M. D., Galesburg, Illinois,.......... 473

### THE CLINIQUE.

CHICAGO MEDICAL DISPENSARY.—Service, Prof. E. ANDREWS, M. D., Attending Surgeon. Calculus,.......... 476

MARINE HOSPITAL.—Dr. ISHAM'S Service. Reported by A. D. ROUSE, Hospital Interne. I. Dislocation of Humerus of Fourteen Weeks. Recovery,.......... 480

Operation for Hernia,.......... 481

"Drill for Auscultation"—No. 1. By THOS. K. CHAMBERS, M. D., etc.,.......... 482

### SELECTIONS.

Renunciation of Homœopathy. By the late Editor of the *North American Journal of Homœopathy*,.......... 488

Wardell on Enteric Fever,.......... 491

Tin Fractured Splints,.......... 475

French Surgery in the Crimea,.......... 494

### BOOK NOTICES.

Lecture on the Diagnosis and Treatment of the Principal Forms of the Paralysis of the Lower Extremities. By E. BROWN-SEQUARD, M. D., F. R. S., etc., etc.,.......... 495

New Periodical, *Buffalo Medical and Surgical Journal and Reporter*....The *Lancet*....Annual Address before Connecticut Medical Society....Commuuications of the Rhode Island Medical Society,.......... 500

Transactions of the Indiana State Medical Society,.......... 501

### EDITORIAL.

The Chicago *Medical Journal* and Lind University,.......... 501

Changes in Medical Colleges in this City,.......... 503

Personal....Renunciation of Homœopathy,.......... 504

Further Experiments with Kerosolene,.......... 505

SPECIAL SELECTIONS: Military Hospital Treatment of Gonorhœa.......... 506

The Surgeons of the New York Eighth,.......... 508

Successful Operation for Double Congenital Cataract at the Age of Eighteen,.......... 510

